# Acute Regulation of Cardiac Metabolism by the Hexosamine Biosynthesis Pathway and Protein O-GlcNAcylation

**DOI:** 10.1371/journal.pone.0018417

**Published:** 2011-04-11

**Authors:** Boglárka Laczy, Norbert Fülöp, Arzu Onay-Besikci, Christine Des Rosiers, John C. Chatham

**Affiliations:** 1 Division of Cardiovascular Disease, Department of Medicine, University of Alabama at Birmingham, Birmingham, Alabama, United States of America; 2 Department of Nephrology, Kaposi Mór County Hospital, Kaposvár, Hungary; 3 Department of Pharmacology, University of Ankara, Ankara, Turkey; 4 Montreal Heart Institute and Department of Nutrition, Université de Montréal, Montreal, Quebec, Canada; Pennington Biomedical Research Center, United States of America

## Abstract

**Objective:**

The hexosamine biosynthesis pathway (HBP) flux and protein O-linked N-acetyl-glucosamine (O-GlcNAc) levels have been implicated in mediating the adverse effects of diabetes in the cardiovascular system. Activation of these pathways with glucosamine has been shown to mimic some of the diabetes-induced functional and structural changes in the heart; however, the effect on cardiac metabolism is not known. Therefore, the primary goal of this study was to determine the effects of glucosamine on cardiac substrate utilization.

**Methods:**

Isolated rat hearts were perfused with glucosamine (0–10 mM) to increase HBP flux under normoxic conditions. Metabolic fluxes were determined by ^13^C-NMR isotopomer analysis; UDP-GlcNAc a precursor of O-GlcNAc synthesis was assessed by HPLC and immunoblot analysis was used to determine O-GlcNAc levels, phospho- and total levels of AMPK and ACC, and membrane levels of FAT/CD36.

**Results:**

Glucosamine caused a dose dependent increase in both UDP-GlcNAc and O-GlcNAc levels, which was associated with a significant increase in palmitate oxidation with a concomitant decrease in lactate and pyruvate oxidation. There was no effect of glucosamine on AMPK or ACC phosphorylation; however, membrane levels of the fatty acid transport protein FAT/CD36 were increased and preliminary studies suggest that FAT/CD36 is a potential target for O-GlcNAcylation.

**Conclusion/Interpretation:**

These data demonstrate that acute modulation of HBP and protein O-GlcNAcylation in the heart stimulates fatty acid oxidation, possibly by increasing plasma membrane levels of FAT/CD36, raising the intriguing possibility that the HBP and O-GlcNAc turnover represent a novel, glucose dependent mechanism for regulating cardiac metabolism.

## Introduction

Cardiovascular complications, including diabetic cardiomyopathy are the leading cause of excessive premature morbidity and mortality in diabetic patients. Maladaptive alterations of cardiac metabolism plays a pivotal role in the development of diabetic cardiomyopathy [1] and abnormal regulation of carbohydrate and fatty acid metabolism has been well-characterized as one of the earliest adverse manifestation of diabetes on cardiac myocyte function [1], [2]. Typically the metabolic shift towards decreased myocardial carbohydrate oxidation and increased fatty acid metabolism that occurs in diabetes is attributed to an increase in circulating lipids via the classical Randle Cycle [3]; however, hearts from young db/db mice exhibited significantly increased fatty acid oxidation and decreased carbohydrate oxidation, prior to the onset of overt hyperglycemia and in the absence of increased circulating lipids [4]. This suggests that metabolic dysfunction occurs prior to the onset of overt diabetes and may be due to mechanisms independent of Randle Cycle.

The modification of proteins by O-linked N-acetylglucosamine (O-GlcNAc) was first identified by Torres and Hart in 1984 [5] and there is a growing evidence implicating excessive O-GlcNAcylation in glucose toxicity and insulin resistance [6], [7], major hallmarks of diabetes mellitus and diabetes-related complications. In contrast to classical protein glycosylation in the ER and Golgi, characterized by stable and complex elongated oligosaccharide structures, O-GlcNAcylation is a dynamic process involving the reversible addition of a single O-GlcNAc moiety to serine and threonine residues of nuclear and cytosolic proteins [8]. This process is regulated by the activities of two key enzymes, O-GlcNAc transferase (OGT), which catalyzes the attachment of O-GlcNAc and N-acetylglucosaminidase (O-GlcNAcase), which catalyzes its removal [8]. The activity of OGT is sensitive to the intracellular concentration of its substrate [8], UDP-GlcNAc, which is the product of the hexosamine biosynthesis pathway (HBP). Flux through the HBP and thus the synthesis of UDP-GlcNAc is regulated in large part by the metabolism of glucose; this is regulated by l-glutamine-d-fructose 6-phosphate amidotransferase (GFAT), which converts fructose-6-phosphate to glucosamine-6-phosphate with glutamine as the amine donor [9].

Given the central role of glucose in regulating HBP flux and thus cellular levels of O-GlcNAc, accumulating evidence suggests that increased protein O-GlcNAcylation due to nutrient excess mediate the adverse effects of diabetes in the cardiovascular system. While much of the work in this area has focused on endothelial-vascular complications [10], [11], [12], increased O-GlcNAc levels have been linked to cardiac myocyte dysfunction seen in diabetes [13], [14], as well as implicated in impaired hypertrophic and alpha-adrenergic signaling [15], [16]. An early consequence of diabetes on the heart is an increased fatty acid oxidation, which has been implicated in diabetes-induced cardiac dysfunction [17], [18]. As noted above, the increase in myocardial fatty acid utilization seen in diabetes has typically been attributed to an increase in circulating lipids and implicated in lipotoxicity, mitochondrial dysfunction and impaired myocardial bioenergetics [1], [2], [17], [18]. However, Luo et al. reported that an increase in O-GlcNAc levels in adipocytes by the activation of the HBP with glucosamine, increased fatty acid oxidation [19], which raises the intriguing possibility that that the HBP and protein O-GlcNAcylation could modulate regulation of substrate metabolism in the heart.

Although it has been shown that acute increases in HBP flux and O-GlcNAc with glucosamine mimicked some of the effects of diabetes on the heart [14], [20], there are no data regarding the role of the HBP and O-GlcNAc in the regulation of cardiac metabolism. Therefore, the goal of this study was to determine the effects of activation of the HBP with glucosamine on cardiac metabolic regulation. We found that glucosamine significantly decreased total carbohydrate oxidation, increased fatty acid oxidation, and this was associated with increased cardiac O-GlcNAcylation. In addition we found that this increase in fatty acid oxidation appeared to be a consequence of increased levels of the fatty acid transporter FAT/CD36 at the plasma membrane rather than alterations in AMPK or ACC activity. Preliminary studies also indicate that FAT/CD36 may be subject to direct O-GlcNAc modification.

## Materials and Methods

### Ethics statement

Animal experiments were approved by the University of Alabama Institutional Animal Care and Use Committee (UAB APN 100408442) and conformed to the Guide for the Care and Usage of Laboratory Animals published by the National Institutes of Health (NIH Publication No. 85-23, 1996).

### Materials

Non-fasted, 300–350 g male Sprague Dawley rats (Charles Rivers Laboratories) were used in all studies. Unless otherwise noted, chemicals were obtained from Fisher Scientific (Santa Clara, CA) or Sigma-Aldrich (St. Louis, MO). Essentially fatty acid free bovine serum albumin was obtained from Serologicals Proteins Inc. (Kankakee, IL). ^13^C-labeled substrates were obtained from Cambridge Isotope Laboratories (Andover, MA).

### Isolated heart perfusions

Animals were anesthetized, hearts rapidly excised and perfused retrogradely at a constant perfusion pressure of 75 mmHg with Krebs-Henseleit buffer containing (in mM) glucose 5.0, lactate 1.0, pyruvate 0.1, palmitate 0.32, glutamine 0.5 and 3% BSA (fatty acid free) plus 50 µU/mL insulin (NovoNordisk), as previously described [21]. Cardiac function was monitored via a fluid-filled balloon placed into the left ventricle and end-diastolic pressure (EDP) was set to 5 mmHg. All hearts were paced continuously at 320 beats/min.

Hearts were assigned to one of six groups and perfused for 60 minutes under normoxic conditions with perfusion buffer containing: 1) 0 mM glucosamine (n = 8); 2) 0.05 mM glucosamine (n = 5); 3) 0.1 mM glucosamine (n = 8); 4) 1.0 mM glucosamine (n = 4); 5) 5.0 mM glucosamine (n = 8); and 6) 10.0 mM glucosamine (n = 7).

The upper concentrations of glucosamine were chosen based on our earlier report demonstrating that perfusion of normal hearts with 5 mM glucosamine mimicked the effects of short-term diabetes on the heart [22] and the study by Luo et al. [19], who reported that 10 mM glucosamine increased fatty acid oxidation in cultured adipocytes. To determine the range of glucosamine concentrations, which modulated fatty acid oxidation, additional experiments were performed at 0.05, 0.1 and 1 mM.

### ^13^C-isotopomer analyses

Hearts were perfused with [U-^13^C]palmitate, [3-^13^C]lactate and [2-^13^C]pyruvate for the final 40 minutes of the protocol at which time hearts were freeze-clamped, acid extracted and ^13^C-NMR isotopomer analysis was performed as previously described [23], [24]. This enabled us to determine the fraction of total acetyl-CoA entering the TCA cycle that originating from unlabeled, [1,2-^13^C]-, [2-^13^C]- and [1-^13^C]acetyl-CoA originating from unlabeled glucose, [U-^13^C]-palmitate, [3-^13^C]-lactate and [2-^13^C]-pyruvate respectively [23], [24]. We have previously shown that under these perfusion conditions in the isolated rat heart, there is negligible contribution from endogenous triglycerides to unlabeled acetyl-CoA formation [25].

### Determination of lactate efflux and uptake rates

^1^H-NMR spectroscopy was used to determine the ratio of unlabeled lactate formed by the exogenous glucose or endogenous glycogen and [3-^13^C]lactate added to the perfusate. These data, multiplied by the total lactate concentration in the effluent and coronary flow, were used to determine the rates of exogenous [3-^13^C]lactate uptake and unlabeled endogenously produced lactate efflux as described in detail elsewhere [23], [24].

### Immunoblot analyses

Tissue was homogenized in the appropriate lysis buffer as previously described for O-GlcNAc [20], [21], and for phospho- and total-ACC and phospho- and total-AMPK [26]. Whole heart lysates were separated on SDS-PAGE and transferred to PVDF membrane (Pall). Membranes were probed for O-GlcNAc (CTD110.6 antibody, a kind gift from Mary-Ann Accavitti, UAB Epitope Recognition and Immunoreagent Core), and for phospho- and total-ACC (Cell Signaling) and phospho- and total-AMPK (Cell Signaling) antibodies. Blots were visualized with enhanced chemiluminescence assay (Pierce) and the signal was detected with UVP BioChemi System (UVP). Densitometry was quantified using Labworks analysis software (UVP).

### Membrane associated FAT/CD36 levels

Tissue powder was lyzed in ice-cold homogenization buffer containing (in mM) Tris (pH: 7.4) 20.0, EDTA 5.0, sucrose 250.0, phenylmethanesulfonyl fluoride 1.0 and 2.5% protease inhibitor cocktail. Tissue homogenates were centrifuged at 1,000 g for 10 min. The resulting supernatant was ultracentrifuged at 110,000 g for 75 min at 4°C. The pellet ( = particulate membrane fraction) was resuspended and incubated for 30 min in ice-cold solubilization buffer containing (in mM) Tris (pH: 7.4) 50.0, NaCl 100.0, LiCl 50.0, EDTA 5.0, and 0.5 (v/v) % Triton X-100, 0.05 (w/v) % SDS, 0.5 (w/v) % sodium deoxycholate, and 0.02 (w/v) % sodium azide. The samples were centrifuged at 14,000 g for 10 min at 4°C, and the supernatant was mixed with reducing sample buffer (BioRad) and boiled for 5 min. Proteins were resolved by SDS-PAGE and blotted on Immobilion-P PVDF membranes (Millipore). The blots were probed with FAT/CD36 antibody (Cascade Biosciences) and visualized as described above. GAPDH and pan-cadherin antibodies (Abcam) were used to verify the purity of the membrane fraction and pan-cadherin was used as a loading control.

### Immunoprecipitation

Cardiac tissue was homogenized to obtain either whole tissue (w) or membrane fraction (m) lysates, as described above. Samples containing equal amount of protein (1000 µg) were mixed with 5 µg of polyclonal rabbit anti-CD36 antibody (sc-9154, Santa Cruz Biotechnology) overnight at 4°C with protein A agarose beads (Upstate). The agarose beads then were washed three times in PBS containing 1% NP-40 followed by washes with PBS for three times. Antigens were eluted from the beads and boiled for 5 min in Laemmli buffer prior to SDS-PAGE. Immunoblots were incubated with CTD 110.6 (1∶1000), anti-CD36 (1∶500), and anti-OGT (DM-17; 1∶1000, Sigma) antibodies for overnight at 4°C, followed by incubation with appropriate secondary antibodies and chemiluminescence visualization.

Control experiments included immunoprecipitation in the absence of samples and/or antibody. The CD36 positive protein band at the appropriate molecular weight (∼88 kD) was only observed in the presence of both antibody and sample (data not shown), demonstrating that this band was indeed CD36 and not a non-specific protein band. The specificity of the O-GlcNAc antibody was also confirmed by co-incubation with 10 mM N-acetylglucosamine [27].

### HPLC analyses

Approximately 50 mg of frozen tissue powder was homogenized in 1 mL ice-cold 0.3 mol/L perchloric acid and centrifuged at 15,000 g for 15 min at 4°C. PCA was removed from the supernatant with 2 volumes of 1∶4 trioctylamine:1,1,2-trichloro-trifluoroethan mixture. Samples were loaded on Partisil 10 SAX column (Beckman), nucleotide sugars were measured at 262 nm using 2 mL/min flow rate and linear salt and pH gradient from 5 mM to 750 mM (NH_4_)H_2_PO_4_ and from pH 2.8 to 3.7 [21].

### Data analysis

Data are presented as means ± standard errors. Differences between experimental groups were evaluated with one-way ANOVA with Dunnett's posthoc test or Student's t-test as indicated in the figure legends. Statistically significant differences between groups were defined as *P<0.05* and are indicated in the legends of figures.

## Results

### Effect of glucosamine on cardiac function

Consistent with earlier studies [21], [28], perfusion of the hearts with 0.05, 0.1, 1.0, 5.0 or 10.0 mM glucosamine for 60 minutes had no effect on rate pressure product, ±dp/dt or coronary flow compared to the control hearts, perfused without glucosamine (Table 1).

**Table 1:** Cardiac function of isolated rat hearts perfused with 0 (n = 8), 0.05 (n = 5), 0.1 (n = 10), 1 (n = 4), 5 (n = 8) and 10 mM (n = 7) glucosamine for 60 minutes (hearts were paced at 320 beats/min rate).

Glucosamine concentration (mM)						
	0	0.05	0.1	1	5	10
**RPP (mmHg/min×10^−3^)**	29.1±1.0	33.2±2.3	28.0±1.1	29.8±2.4	28.7±1.5	31.1±1.3
**+dp/dt (mmHg/s×10^−3^)**	4.1±0.2	4.1±0.3	3.5±0.1	4.0±0.4	3.6±0.2	a3.6±0.2
**−dp/dt (mmHg/s×10^−3^)**	2.1±0.1	2.2±0.2	1.8±0.1	2.0±0.2	1.9±0.1	2.1±0.3
**Coronary flow (ml/min)**	11.5±0.8	12.2±0.6	11.1±0.4	11.5±0.7	12.5±0.6	13.5±0.9

Glucosamine had no effect either on cardiac or on coronary flow. (RPP: rate pressure product = left ventricular developed pressure×heart rate; dp/dt: change of pressure over time).

### UDP-GlcNAc and O-GlcNAc levels

**Figure 1:**
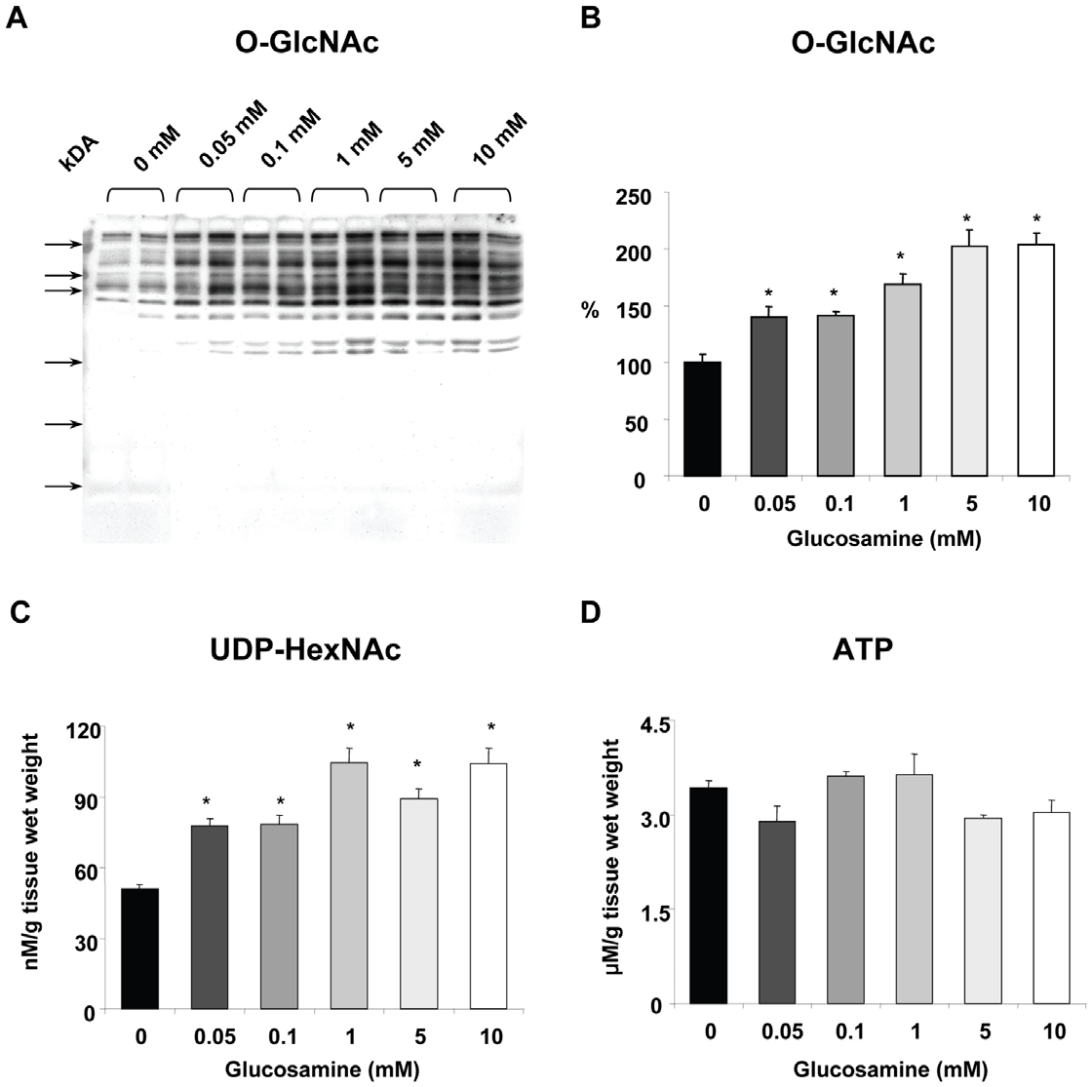
Effect of glucosamine on A, B) Overall cardiac O-GlcNAc levels; C) UDP-HexNAc concentrations and D) ATP concentrations. ^*^ P<0.05 vs. 0 mM, one-way ANOVA with Dunnett's posthoc test. Western blots: 0 mM (n = 8), 0.05 mM (n = 5), 0.1 mM (n = 9), 1 mM (n = 4), 5 mM (n = 8), 10 mM (n = 7). HPLC: 0 mM (n = 4), 0.05 mM (n = 5), 0.1 mM (n = 5), 1 mM (n = 4), 5 mM (n = 3), 10 mM (n = 3). Note that equal protein loading for the O-GlcNAc immunoblots was assessed by Sypro staining and overall O-GlcNAc levels were normalized to untreated control group.

Protein O-GlcNAc levels assessed by immunoblot analysis indicated a clear dose response with glucosamine (Figures 1A, B). Perfusion for 60 min with as little as 0.05 mM glucosamine resulted in ∼40% increase in O-GlcNAc levels and a maximal 2-fold increase in O-GlcNAc was seen with 5 mM glucosamine (Figures 1A, B). Sypro staining was used to ensure equal protein loading for the O-GlcNAc immunoblots; overall O-GlcNAc levels were assessed by densitometric analyses and normalized to untreated control group. Note that Figure 1A is a representative image, with 2 samples for each concentration, whereas the mean data in Fig. 1B is from an n of 3–9 for each concentration as indicated in the figure legend.

UDP-GlcNAc levels exhibited similar response to glucosamine perfusions as O-GlcNAc levels; there was a significant increase with 0.05 mM glucosamine and a maximal ∼2-fold increase with 1 mM (Figure 1C). Others have reported that in cell culture studies glucosamine treatment was associated with decreased ATP levels [29]; however, here in the isolated perfused heart we found that there was no effect of glucosamine on ATP (Figure 1D).

### Cardiac substrate utilization

**Figure 2:**
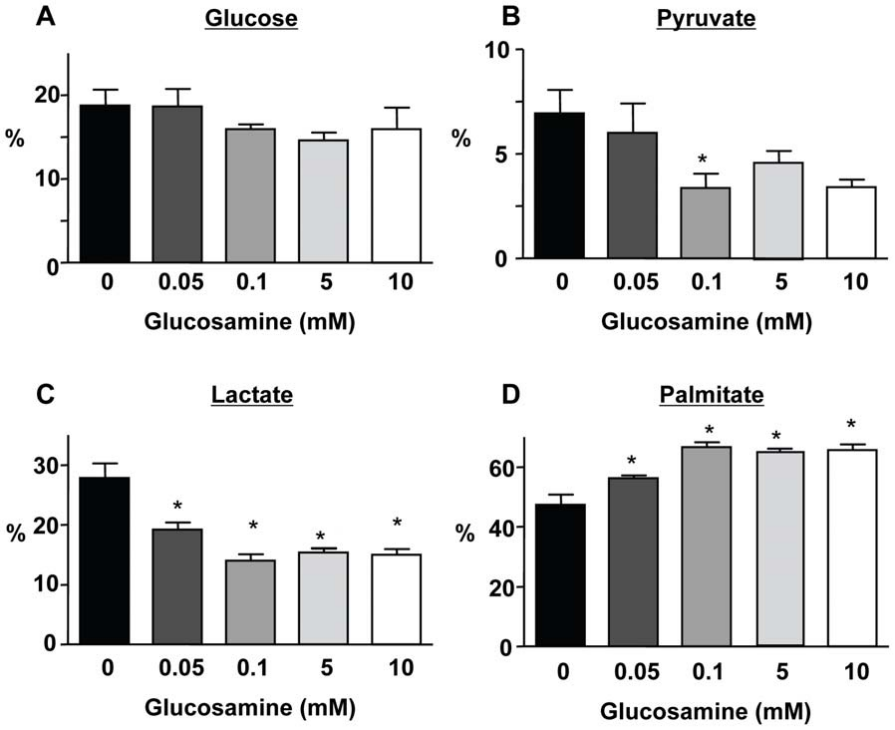
Effect of glucosamine on A) glucose; B) pyruvate; C) lactate and D) palmitate oxidation. ^*^ P<0.05 vs. 0 mM glucosamine, one-way ANOVA with Dunnett's posthoc test. 0 mM (n = 6), 0.05 mM (n = 4), 0.1 mM (n = 5), 5 mM (n = 5), 10 mM (n = 4).

**Figure 3:**
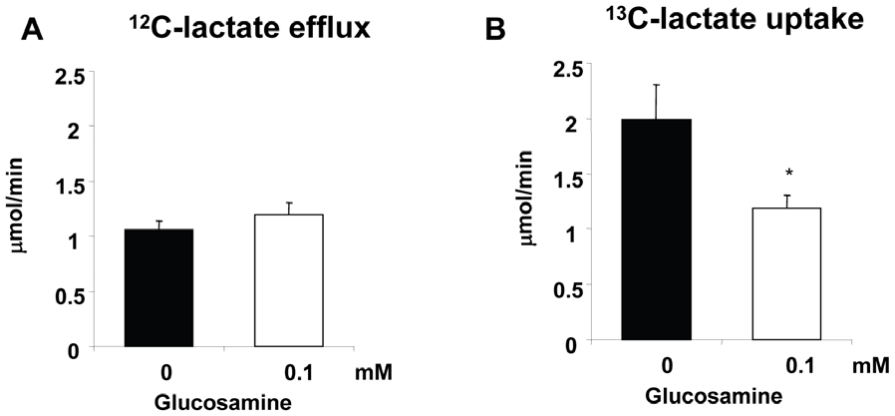
Effect of 0.1 mM glucosamine on A) unlabeled glycolytic lactate efflux and B) exogenous [3-^13^C]lactate uptake ^*^ P<0.05 vs. 0 mM, Student's t-test. 0 mM (n = 4), 0.1 mM (n = 5).

Hearts were perfused with [U-^13^C]palmitate, [3-^13^C]lactate, [2-^13^C]pyruvate and unlabeled glucose; under the conditions of these experiments there is negligible contribution from endogenous triglycerides to unlabeled acetyl-CoA formation [25]. Therefore, the relative contribution of glucose to total substrate entry into the TCA cycle is determined from the fraction of unlabeled acetyl-CoA. Since glucosamine was not enriched with carbon-13, any metabolism of glucosamine via glycolysis would be reflected in an increase in glucose oxidation or glycolysis. However, glucosamine had no effect on the relative contribution of glucose to overall substrate oxidation (Fig. 2A) or the rate of unlabeled glycolytic lactate efflux (Figure 3A). Surprisingly, however, glucosamine significantly decreased both pyruvate and lactate oxidation (Figures 2B, C) and this was associated with a concomitant increase in palmitate oxidation (Figure 2D). The effect of glucosamine on lactate and palmitate oxidation was apparent with as little as 0.05 mM glucosamine and was maximal at 0.1 mM. Consistent with the ∼2-fold decrease in lactate oxidation, glucosamine decreased exogenous ^13^C-labeled lactate uptake rates by ∼2-fold (Figure 3B). This is in agreement with previous studies, showing that the primary metabolic fate of exogenous lactate uptake was subsequent oxidation [30].

### AMPK and ACC phosphorylation

**Figure 4:**
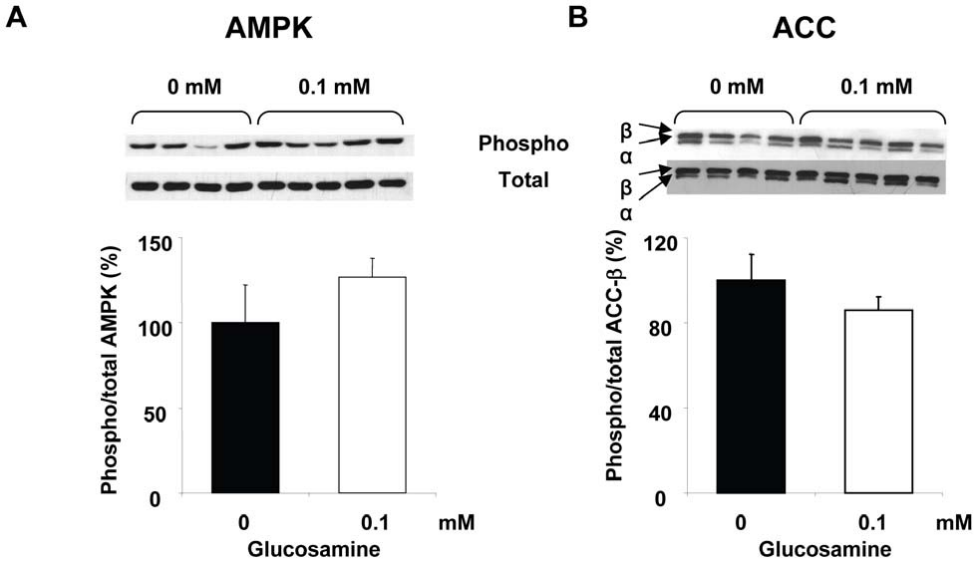
A) AMPK and B) ACC phosphorylation in the heart after 60 min perfusion with 0.1 mM glucosamine. 0 mM (n = 4), 0.1 mM (n = 5).

Since glucosamine increased AMPK activity and ACC phosphorylation in adipocytes [19] we examined the effects of glucosamine on AMPK and ACC phosphorylation in heart. AMPK phosphorylation levels were not significantly altered at any glucosamine concentration (data not shown); since a maximal effect on fatty acid oxidation was seen with 1 mM glucosamine we then compared only the untreated control and 0.1 mM glucosamine groups (Figure 4A). While phospho-AMPK levels appear to be modestly elevated in the 0.1 mM group, this was not statistically significant, although, there was greater variance in the control group due to one outlier. However excluding this outlier did not change the conclusions, in large part because in addition to reducing the variance in the control group it also decreased the differences between the two means. In Fig. 4B we compared ACC phosphorylation between untreated control and 0.1 mM glucosamine groups; again there is no significant difference between the two groups. As with AMPK there was one outlier in the control group; however, excluding this from the analysis also did not alter the conclusions. While not significant ACC phosphorylation levels were modestly decreased in the 0.1 mM glucosamine group; interestingly in 5 and 10 mM glucosamine groups there was a significant ∼30% decrease in phospho-ACC levels (data not shown). While this suggests that glucosamine may affect ACC phosphorylation, it should be noted that a lower ACC phosphorylation would typically be associated with decreased rather than increased fatty acid oxidation as seen here.

### Membrane associated FAT/CD36 levels

**Figure 5:**
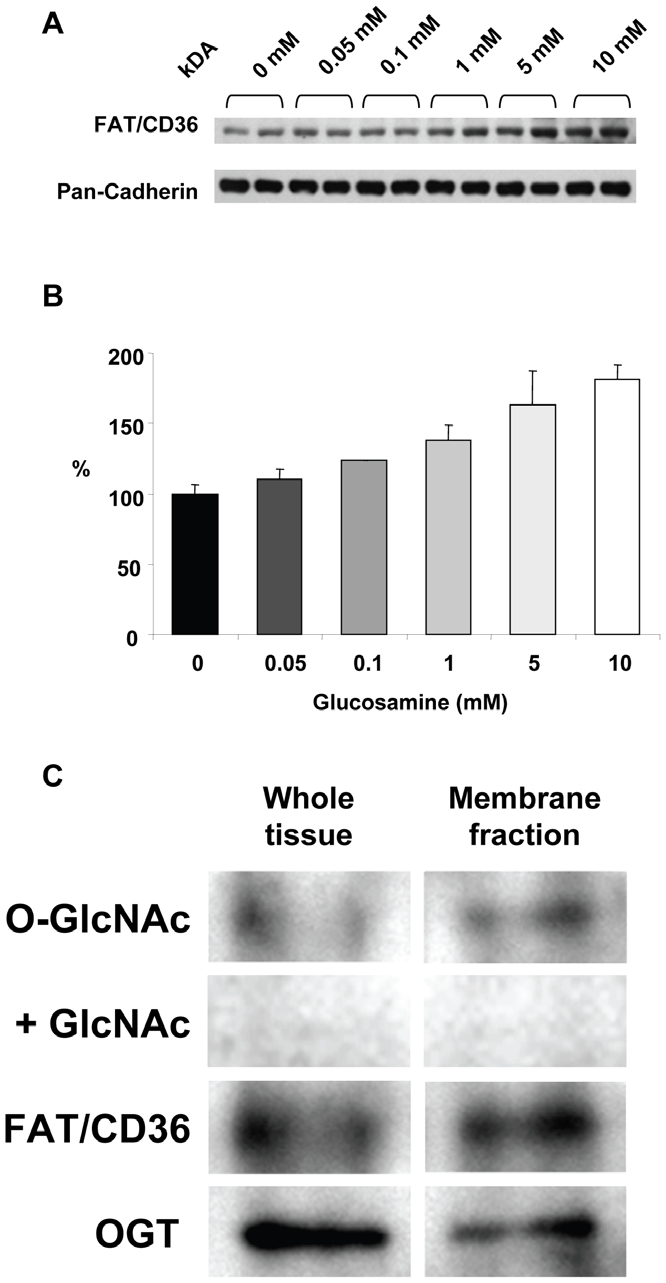
A) Immunoblots of Plasma membrane fraction for FAT/CD36 following 60 min perfusion with 0, 0.05, 0.1, 1, 5 and 10 mM; pan-cadherin included as a plasma membrane marker and protein loading control; B) Densitometric analysis of FAT/CD36 immunoblots normalized to 0 mM glucosamine; P<0.05 vs. 0 mM, one-way ANOVA with Dunnett's posthoc test; *n = 2 in each group*; C) Immunoprecipitation of FAT/CD36 from whole tissue and plasma membrane lysates, followed by O-GlcNAc and OGT immunoblots. Specificity of O-GlcNAc antibody was confirmed by co-incubation with 10 mM N-acetylglucosamine (GlcNAc).

Fatty acid transport into the heart is regulated by membrane levels of FAT/CD36 [31], [32]; therefore, we determined the effect of glucosamine on membrane levels of FAT/CD36. As seen in Figures 5A, B glucosamine treatment markedly increased FAT/CD36 levels in the membrane fraction in a concentration dependent manner; ANOVA indicated that there was a significant treatment effect of glucosamine.

To determine whether the effect of glucosamine on membrane levels of FAT/CD36 could be a result of direct O-GlcNAc modification, FAT/CD36 was immunoprecipitated from both whole tissue and plasma membrane fractions. Since O-GlcNAcylation is known to be a relatively low abundance modification, these studies were performed on hearts where overall O-GlcNAc levels were increased ∼3 fold by perfusion with a combination of glucosamine and O-GlcNAcase inhibition (data not shown). As shown in Figure 5C, there is evidence of O-GlcNAc modification of FAT/CD36 in both fractions; however, this is particularly apparent in the membrane fraction. The lack of signal with the co-incubation of 10 mM N-acetylglucosamine indicates that the positive staining is a result specific binding of the O-GlcNAc antibody [27]. We also found that O-GlcNAc transferase (OGT), the protein responsible for catalyzing the attachment of O-GlcNAc to proteins co-immunoprecipitates with FAT/CD36.

## Discussion

There is a growing appreciation of the importance of protein O-GlcNAcylation as a key regulator of numerous biological processes, including nuclear transport, translation and transcription, signal transduction, proteasomal degradation and apoptosis [8], [33], [34]. Most of our understanding of the role of O-GlcNAcylation on cellular function is in the context of chronic diseases, including diabetes and increased O-GlcNAc levels have been associated with the adverse effects of hyperglycemia and diabetes on the heart [7], [13], [14], [35], [36]. We show here, that acute activation of the HBP in the isolated perfused rat heart with glucosamine significantly decreased total carbohydrate oxidation, increased fatty acid oxidation, and this was associated with increased levels of O-GlcNAcylation. We also show that in contrast to studies in adipocytes [19], this glucosamine-induced increase in fatty acid oxidation appears to be due to increased levels of the fatty acid transporter FAT/CD36 at the plasma membrane rather than alterations in AMPK or ACC activity. Preliminary studies also indicate that FAT/CD36 may be subject to direct O-GlcNAc modification.

The primary pathway for glucosamine metabolism is via the HBP leading to the synthesis of UDP-GlcNAc, which is the precursor for numerous glycosylation reactions in the ER and Golgi as well as the attachment of O-GlcNAc to serine and threonine residues of nuclear and cytoplasmic proteins catalyzed by OGT [8]. In principle, there are also pathways for glucosamine metabolism, which could result increased glycolytic flux [37], [38]; however, glucosamine had no effect on either glucose oxidation or glucose-derived lactate efflux, i.e., glycolysis (Figure 3). Thus, in the heart at least, we found no evidence of metabolism of glucosamine via glycolysis. In contrast to the lack of effect on glucose metabolism, we found that glucosamine significantly increased fatty acid oxidation, with a concomitant decrease in overall carbohydrate oxidation due to lower lactate and pyruvate oxidation (Figure 2). Furthermore, the effect of glucosamine on substrate oxidation was apparent at a glucosamine concentration as low as 0.05 mM with a maximal response at 0.1 mM, which was associated with significant increases in both UDP-GlcNAc and O-GlcNAc levels (Figure 1). These data not only support the notion that the effect of glucosamine on fatty acid oxidation is mediated via the HBP and the subsequent increase in O-GlcNAc levels but also suggest that relatively subtle changes in HBP flux could play an important regulatory role in cardiac metabolism.

Even though concentrations of glucosamine, up to 10 mM, had no further effect on fatty acid or carbohydrate oxidation beyond that seen at 0.1 mM, there was a progressive increase in both UDP-GlcNAc and O-GlcNAc levels. This is consistent with the notion that the primary pathway for glucosamine metabolism in the heart is via the HBP and that over the relatively short time course of these experiments the principal outcome is an increase in O-GlcNAc levels. It is also worth noting that in contrast to previous studies, which indicated that high doses of glucosamine causes a decrease in ATP levels [29], we found that glucosamine had no effect on ATP levels at any concentration (Figure 1D). Furthermore, glucosamine had no adverse effect on cardiac function for the duration of these experiments (Table 1).

While Luo et al., reported that glucosamine stimulated fatty acid oxidation in cultured adipocytes [19], their studies focused on treatment with high glucosamine concentrations (10 mM) over fairly long time period (i.e. 24 hrs). In contrast, here we show in the heart that as little as 0.05 mM glucosamine altered substrate regulation in less than 1 hour with a maximal response at 0.1 mM glucosamine. In adipocytes the glucosamine-induced increase in fatty acid oxidation appeared to be mediated via an O-GlcNAc dependent increase in AMPK activity leading to increased ACC phosphorylation [19]. Although AMPK also plays a key role in the regulating energy metabolism in the heart [39], [40], in contrast to adipocytes [19], we found that 0.1 mM glucosamine had no effect on either AMPK or ACC phosphorylation, which could account for the increase in fatty acid oxidation (Figure 4). Higher concentrations of glucosamine had no effect of AMPK phosphorylation, but at 5 and 10 mM we found significantly decreased ACC phosphorylation (data not shown); however, this would typically be associated with decreased rather than increased fatty acid oxidation.

Another potential mechanism for regulating cardiac fatty acid metabolism is via plasma membrane levels of fatty acid transporter proteins such as FAT/CD36 [41], which is responsible for ∼50–80% of the fatty acid uptake in the heart [31], [32]. Similar to the glucose transporter GLUT4, FAT/CD36 translocates from intracellular storage compartments to plasma membrane in response to stimuli such as insulin or increased cardiac work, thereby facilitating increased fatty acid oxidation [42], [43]. Therefore, we examined FAT/CD36 protein levels in plasma membrane preparations and found that they increased in a dose dependent manner with glucosamine treatment (Figures 5A, B). Preliminary studies also indicated that FAT/CD36 appears to be a target for O-GlcNAc modification and this was supported by the fact that OGT, which catalyzes the attachment of O-GlcNAc co-immunoprecipitated with FAT/CD36 (Figure 5C). Thus, the glucosamine-induced increase in palmitate oxidation may be due, at least in part, to increased plasma membrane levels of FAT/CD36 possibly mediated by increased O-GlcNAc modification of FAT/CD36. While we did not measure rates of fatty acid transport across the plasma membrane, previous studies have demonstrated a close relationship between membrane levels of FAT/CD36 and rates of fatty acid transport [44]. In the future it will be important to determine whether acute pharmacological increases in overall tissue O-GlcNAc levels also lead to increased O-GlcNAcylation of FAT/CD36 and demonstrate that there is a relationship between increased FAT/CD36 O-GlcNAc levels and increased rates of fatty acid transport.

Increased HBP flux has been reported to induce insulin resistance in muscle, which could also lead to an increase in fatty acid oxidation. While we cannot rule out the possibility of decreased insulin sensitivity in this study, the fact that we observed no effect of glucosamine on glycolytic flux or glucose oxidation, suggests that this was not a contributing factor to the increase in fatty acid oxidation. Interestingly, the shift in cardiac metabolism, seen here with glucosamine, namely increased fatty acid oxidation, with decreased carbohydrate oxidation primarily as a result of lower lactate oxidation, is very similar to that previously reported in the diabetic heart perfused under similar conditions [23]. Furthermore, the increase in myocardial fatty acid oxidation seen with diabetes has been linked to increased fatty acid transport and plasma membrane FAT/CD36 expression [43]. In diabetes, the increased sarcolemmal abundance of FAT/CD36 has been shown to be a result of impaired recycling between intracellular storage compartments and the sarcolemma [43]. It is relatively well established that diabetes also increases overall cardiac O-GlcNAc levels [14], [20]; it is possible, therefore, that in the diabetic heart O-GlcNAcylation of FAT/CD36 may shift the balance towards sarcolemmal localization of FAT/CD36 possibly by inhibiting recycling. Clearly future studies are warranted to determine whether O-GlcNAc levels of FAT/CD36 are indeed increased in diabetes and if so, whether this is a contributing factor in the increased levels of sarcolemmal FAT/CD36. For example, determine whether acute overexpression of O-GlcNAcase, which has been shown reverse some of the adverse effects of diabetes on cardiac function [14], would also reduce sarcolemmal FAT/CD36 levels thereby reverse the metabolic dysfunction.

It is noteworthy that while the increase in fatty acid oxidation was maximal at 0.1 mM, FAT/CD36 levels continued to increase up to 5–10 mM glucosamine, similar to the increase in O-GlcNAc levels. The dissociation between increased membrane levels of FAT/CD36 and fatty acid oxidation is consistent with studies in skeletal muscle from obese rats where excess fatty acid uptake was channeled primarily to esterification rather than oxidation [45]. This raises the intriguing possibility that the increased O-GlcNAc levels seen in the heart in response to diabetes could be a contributing factor to the accumulation of lipid intermediates that have been implicated in lipotoxicity. It should be noted however, that we did not assess the effects of glucosamine on triglyceride levels; nevertheless, these results suggest that further studies are warranted to elucidate the effects of the HBP and O-GlcNAcylation on the partitioning of fatty acids between oxidative and non-oxidative metabolic pathways in the heart.

While these data here are consistent with the notion that the effects of glucosamine on myocardial substrate utilization are mediated via increased O-GlcNAc levels a definitive cause and effect relationship has not been shown. In the first instance it is possible that an increase in HBP intermediates could influence carbohydrate and fatty acid oxidation via mechanisms other than increased O-GlcNAc levels. For example, increased glucosamine-6-phosphate levels inhibited hexokinase and stimulated glycogen synthase activity [46]; however, we found no effect of glucosamine on lactate efflux rates (Fig. 3A), which suggests that glycolysis was unaffected. Furthermore, the primary impact of glucosamine on carbohydrate oxidation was at the level of lactate and pyruvate oxidation, also supporting the notion that in these experiments glucosamine had no direct effect on glucose metabolism.

A more definitive approach to demonstrate that OGT is the primary mediator of the effects of glucosamine would to be to show that its effects were blocked by pharmacological inhibition of OGT. Recently novel and potent OGT inhibitors have been described [47], and their effectiveness in decreasing O-GlcNAc levels in isolated neonatal cardiomyocytes has been reported [48]; however, in the intact heart 5 µM of one such inhibitor, TT04, resulted in a marked decline in cardiac function (Chatham, unpublished data). In the future, studies utilizing the recently described cardiac specific OGT KO mouse [49] could also provide valuable insights into the role of O-GlcNAc in the regulation of cardiac metabolism. However it is of note that in isolated cardiomyocytes increasing OGT expression typically mimics the acute effects of glucosamine treatment [50], [51], which is also consistent with concept that that the primary mechanism for mediating the short-term effects of glucosamine is via an increase in flux through OGT. An alternative approach that is commonly used to increase O-GlcNAc levels independent of the HBP is to inhibit O-GlcNAcase; however, earlier studies were limited due to relatively low specificity of available inhibitors. Recently, new highly specific inhibitors have been described [52] and when more widely available would also be valuable tools for elucidating the mechanisms by which protein O-GlcNAcylation influences metabolic regulation.

Our current understanding of cardiac metabolic regulation is primarily based on substrate availability and the effects of hormones, particularly insulin, on phosphorylation of key regulatory proteins including IRS1/2 and AMPK. Increased O-GlcNAcylation has been implicated in the development of insulin resistance increasing adipocyte lipid metabolism by direct modification of IRS1 [53] and AMPK [19] respectively; however, such results have been under conditions of sustained increases in HBP flux and O-GlcNAcylation. Here we show for the first time that glucosamine acutely increases cardiac fatty acid oxidation at relatively low concentrations, and this was associated by an increase in overall O-GlcNAc levels. Furthermore, there was also a dose dependent, glucosamine-induced increase in plasma membrane levels of FAT/CD36 as well as data suggesting that FAT/CD36 is a potential target for O-GlcNAcylation. Clearly further studies are needed to elucidate the specific mechanism(s) associated with the effects of glucosamine on cardiac metabolism; nevertheless, these data raise the intriguing possibility that the HBP and O-GlcNAc turnover represent a novel, glucose dependent mechanism for the acute regulation of cardiac metabolism. Since the metabolic shift seen with glucosamine is similar to that seen with diabetes and given that diabetes leads to chronically elevated O-GlcNAc levels, this may also represent another mechanism contributing to the metabolic inflexibility that is characteristic of the effects of diabetes on the heart.
